# Tollip deficiency exaggerates airway type 2 inflammation in mice exposed to allergen and influenza A virus: role of the ATP/IL-33 signaling axis

**DOI:** 10.3389/fimmu.2023.1304758

**Published:** 2023-12-06

**Authors:** Hamid Reza Nouri, Niccolette Schaunaman, Monica Kraft, Liwu Li, Mari Numata, Hong Wei Chu

**Affiliations:** ^1^ Department of Medicine, National Jewish Health, Denver, CO, United States; ^2^ Department of Medicine, Icahn School of Medicine at Mount Sinai, New York, NY, United States; ^3^ Department of Biological Sciences, College of Science, Virginia Tech, Blacksburg, VA, United States

**Keywords:** extracellular ATP, IL-33, influenza A virus, Th2 inflammation, Tollip deficiency

## Abstract

Toll-interacting protein (Tollip) is a negative regulator of the pro-inflammatory response to viruses, including influenza A virus (IAV). Genetic variation of Tollip has been associated with reduced airway epithelial Tollip expression and poor lung function in patients with asthma. Whether Tollip deficiency exaggerates type 2 inflammation (e.g., eosinophils) and viral infection in asthma remains unclear. We sought to address this critical, but unanswered question by using a Tollip deficient mouse asthma model with IAV infection. Further, we determined the underlying mechanisms by focusing on the role of the ATP/IL-33 signaling axis. Wild-type and Tollip KO mice were intranasally exposed to house dust mite (HDM) and IAV with or without inhibitors for IL-33 (i.e., soluble ST2, an IL-33 decoy receptor) and ATP signaling (i.e., an antagonist of the ATP receptor P2Y13). Tollip deficiency amplified airway type 2 inflammation (eosinophils, IL-5, IL-13 and mucins), and the release of ATP and IL-33. Blocking ATP receptor P2Y13 decreased IL-33 release during IAV infection in HDM-challenged Tollip KO mice. Furthermore, soluble ST2 attenuated airway eosinophilic inflammation in Tollip KO mice treated with HDM and IAV. HDM challenges decreased lung viral load in wild-type mice, but Tollip deficiency reduced the protective effects of HDM challenges on viral load. Our data suggests that during IAV infection, Tollip deficiency amplified type 2 inflammation and delayed viral clearance, in part by promoting ATP signaling and subsequent IL-33 release. Our findings may provide several therapeutic targets, including ATP and IL-33 signaling inhibition for attenuating excessive airway type 2 inflammation in human subjects with Tollip deficiency and IAV infection.

## 1 Introduction

Acute exacerbations of asthma are associated with respiratory viral infections, which continue to pose a significant burden for patients, healthcare providers, and the economy (1). Respiratory viruses contribute to 80% and 50% of asthma exacerbations in children and adults, respectively (2, 3). Rhinovirus and respiratory syncytial virus (RSV) are commonly detected in asthma exacerbations, but influenza A virus (IAV) infections in asthma patients have been linked to more severe exacerbations (4). It was reported that asthma was one of the most underlying medical conditions among hospitalized patients due to influenza A(H1N1)pdm09 viral infection in 2009 (5). In addition, children with asthma were more susceptible to IAV infection during the 2009-2010 flu season (6). Children who developed acute pneumonia as a result of pH1N1 infection had elevated levels of type 2 (T2) cytokines in the serum (7). The mechanisms by which IAV induces asthma exacerbations are still unclear, although viral exacerbations of asthma have been associated with increased pro-inflammatory cytokines, disruption of the epithelial barrier, impaired apoptosis and decreased IFN-γ production (8).

Toll-interacting protein (Tollip) is a multifunctional immune regulator that has been implicated in several lung diseases (9). Tollip is constitutively expressed by many types of cells, including macrophages and epithelial cells. The classical functions of Tollip include inhibition of pro-inflammatory response induced by Toll-like receptor (TLR) agonists and pathogen infections (10–12). However, recent studies have discovered the non-classical functions of Tollip, such as autophagy and stabilization of antiviral proteins (13, 14). In humans, a Tollip single nucleotide polymorphism (SNP) has been associated with decreased Tollip gene expression and increased susceptibility to idiopathic pulmonary fibrosis (15). Our group has shown that Tollip rs5743899 is associated with reduced Tollip expression in human airway epithelial cells and airflow limitation in asthma subjects (16).

The role of Tollip in viral exacerbations of asthma remains unclear. Our previous study suggests that Tollip deficiency enhanced lung non-type 2 (e.g., neutrophilic) inflammation, which was coupled with increased extracellular ATP and IL-33 release (17). However, whether Tollip regulates type 2 inflammation during IAV infection via extracellular ATP and IL-33 has not been investigated. IL-33 is a member of the IL-1 family, which can be increased in asthma patients and released during virus-induced exacerbations (18, 19). *IL-33 through binding to the membrane bound receptor ST2L stimulates* Th2, mast cells *and group 2 innate lymphoid cells (ILC2s) to release* type 2 (T2) cytokines, such as *IL-13 and IL-5* (20), which in turn regulate type 2 inflammation including eosinophil recruitment and survival, and mucus production (21).

Mice treated with house dust mite (HDM) and IAV increased IL-33 production (22), but how IL-33 is released during IAV infection in lungs with allergic inflammation remains unclear. Upon exposure to airborne allergens or viral infection (23, 24), mitochondrial damage (25) and associated adenosine 5′-triphosphate (ATP) secretion (26) occur. It has been proposed that increased extracellular ATP via binding and activating the ATP receptor P2Y13 may lead to the release of processed/activated IL-33 (27). As Tollip is an essential coordinator to remove damaged mitochondria (28), it likely regulates ATP release, but this has not been studied.

In the current study, we hypothesized that Tollip deficiency exaggerates type 2 inflammation during IAV infection by increasing ATP release to the extracellular space, which utilizes an ATP receptor P2Y13 to increase IL-33 release. To test our hypothesis, we utilized a Tollip deficient mouse model of house dust mite (HDM) challenges and IAV infection in the presence and absence of a highly selective pharmacological inhibitor of P2Y13, and sST2, a decoy IL-33 receptor.

## 2 Materials and methods

### 2.1 Preparation of IAV

The pandemic influenza A/California/07/2009 (CA07) virus (29) used in this study was provided by Dr. Mari Numata from National Jewish Health (NJH). Briefly, IAV was propagated in Madin-Darby canine kidney (MDCK; ATCC, Manassas, VA, USA) cells in Dulbecco’s Modified Eagles Medium (DMEM; Thermo Fisher Scientific, Waltham, MA, USA), supplemented with 10% fetal calf serum (MilliporeSigma, Burlington, MA, USA), L-glutamine, penicillin-streptomycin, and 1.5 μg/mL of N-tosyl-Lphenylalanine chloromethyl ketone (TPCK)-treated trypsin (Thermo Fisher Scientific, Waltham, MA, USA), harvested at 72 hour post-infection, and tittered by quantitative plaque assay using MDCK cells as previously reported (29).

### 2.2 Murine models of house dust mite-induced lung inflammation and IAV infection

Tollip knockout (KO) mice on C57/BL6 background were obtained from Dr. Liwu Li at Virginia Polytechnic Institute and State University and bred at the NJH Biological Resource Center (BRC). Wild-type (WT) C57BL/6 mice were purchased from the Jackson Laboratory (Bar Harbor, ME, USA). Mice were housed at NJH BRC under pathogen-free housing conditions and maintained on a 12-hour light-dark cycle with access to food and water. The Institutional Animal Care and Use Committee at NJH approved all the experiments. Groups of Tollip KO and WT mice (8–12 weeks old, both females and males) with age and gender matched, were assigned to four treatment groups, phosphate buffered saline (PBS) as control, HDM, IAV and HDM+IAV. Mice were lightly anesthetized with isoflurane, then intranasally exposed to 10 μg of HDM (30) or PBS on days 0 and 7, and then challenged daily with HDM at days 14–16. Two days after the last HDM challenge, mice were inoculated intranasally with 1x10^2^ pfu/mouse of IAV in 50 µL of PBS or 50 µL of PBS (17). Mice were sacrificed at day 7 and day 10 post IAV infection (**Supplementary Figure 1**).

### 2.3 Soluble ST2 (sST2) treatment during IAV infection in HDM-challenged mice

Using the HDM and IAV infection model as described above, Tollip KO and WT mice were intranasally inoculated with 10 µg of recombinant mouse ST2 (R&D Systems, Minneapolis, MN, USA), or IgG control (17), in 50 µL of PBS on day 8 and day 9 post IAV infection. Mice were sacrificed on day 10 post infection.

### 2.4 Treatment of a selective ATP receptor P2Y13 antagonist during IAV infection in HDM-challenged and IAV-infected mice

Using the HDM and IAV infection model as described above, Tollip KO mice were **intraperitoneally** treated with 50 µl of PBS (control) or a highly selective ATP receptor P2Y13 antagonist MRS2211 (TOCRIS, USA) (31) at 1 mg/kg body weight after one hour of IAV infection, and then once daily on days 1, 4, 5 and 6 post infection. Mice were sacrificed on days 7 and 10 post infection. The dose of MRS2211 was chosen based on our pilot dose optimization study and previously published research (27).

### 2.5 Mouse bronchoalveolar lavage fluid and lung tissue processing

Mice were euthanized by intraperitoneal injection of pentobarbital sodium (Fatal-Plus). BAL was performed with 1 mL of sterile saline. Cell-free BAL fluid (BALF) was used for ELISA, western blot analysis, and ATP measurement. Cytospin slides prepared from BAL cells were stained with a Diff-Quick stain kit (IMEB, San Marcos, CA, USA) for cell differential counts. Leukocyte differentials were determined as a percentage of 500 counted leukocytes. Left lungs were saved in RLT (QIAGEN, MD, USA) or radioimmunoprecipitation assay (RIPA) lysis buffer at -20°C for gene or protein expression assays, respectively. The apical lobe of the right lung was fixed in 10% formalin, embedded in paraffin, and cut at 5-µm thickness for histopathologic evaluation and mucin staining.

### 2.6 Mouse lung histopathologic analysis and mucin staining

Lung tissue sections were stained with hematoxylin and eosin (H&E), and assessed blindly under a light microscope using the histopathological inflammatory scoring system as described previously (32). Briefly, each tissue section was evaluated based on five categories: a) the number of bronchiolar and bronchial sites with inflammatory cells infiltrate, b) the severity of infiltrate of inflammatory cells, c) the severity of bronchiolar and bronchial luminal exudate, d) the frequency of perivascular inflammatory cells infiltrate, and e) the severity of parenchymal pneumonia. In addition, airway mucin production was identified by PAS staining and analyzed. Medium-sized airways were examined for quantifying airway mucins (33, 34). The area of mucins in the epithelium of five complete airways per mouse was evaluated using the NIH ImageJ software (National Institutes of Health, Bethesda, MD). The results were expressed as airway mucin area/total airway epithelium area (percentage).

### 2.7 Western blot analysis

IL-33 released into mouse BALF was measured via western blot as previously described (17). An equal volume of BALF (25 µl) was run on 15% polyacrylamide gels, and proteins were separated via denaturing sodium dodecyl sulfate-polyacrylamide gel electrophoresis (SDS-PAGE). Briefly, proteins were transferred to a polyvinylidene difluoride (PVDF) membrane (Millipore, USA), blocked with 5% skimmed milk (Sigma, USA), and incubated with a goat anti-mouse IL-33 antibody (1:500, AF3626; R&D Systems, Minneapolis, MN, USA) overnight at 4°C, followed by detection with a horseradish peroxidase-conjugated secondary antibody against goat (1:3000, HAF017; R&D Systems, Minneapolis, MN, USA). The signal was developed using the chemiluminescent method recommended by the manufacturer (ECL, USA) and captured using a Fotodyne imaging system (Fotodyne, Inc., Hartland, WI, USA). Western blotting of BALF soluble E-cadherin - an indicator of lung epithelial integrity or injury was performed. Briefly, an equal volume of mouse BALF was run on 8% polyacrylamide gels, and probed with a goat anti-mouse E-cadherin antibody (R&D Systems). The densitometry values in equal volumes (25 µl) of BALF were used to indicate IL-33 or E-cadherin levels. Protein levels in the control group of WT mice were used to normalize those in other groups of mice.

### 2.8 Measurements of adenosine triphosphate, lactate dehydrogenase and cytokines

ATP in BALF was assessed using a Fluorometric Assay Kit (SIGMA ALDRICH, MO, USA). The LDH detection kit (Roche Diagnostics, Indianapolis, IN, USA) was used to assess the cytotoxic effect of different treatments. Data were expressed as the fold changes of different groups versus the control groups. Mouse CXCL1/KC ELISA kit (R&D Systems Inc. Minneapolis, MN, USA) was used to quantify KC in BALF.

### 2.9 Quantitative real-time PCR

TaqMan real-time PCR assays including primers and probes were purchased from ThermoFisher (Waltham, MA, USA) to assess mRNA expression of IL-5 (assay ID: Mm00439646_m1), IL-13 (assay ID: Mm00434204_m1) and Muc5ac (assay ID: Mm01276718_m1). Lung tissue INF-β and IAV expression was measured with custom-made primers and probe (Integrated DNA Technologies, Coralville, IA). Briefly, specific primers and probes used to amplify INF-β gene are: forward primer: 5′-GACGGAGAAGATGCAGAAGAG-3′, reverse primer: 5′-CCACCCAGTGCTGGAGAA-3′, probe: 5′-FAM/TGCCTTTGCCATCCAAGAGAT-3′. Specific primers and probes (17) were used to amplify M1 protein of IAV: forward primer: 5′-GACCRATCCTGTCACCTCTGAC-3′, reverse primer: 5′-AGGGCATTYTGGACAAAKCG TCTA-3′, probe: 5′- FAM/TGCAGTCCTCGCTCACTGGGCACG-3′. Target gene expression was normalized to the housekeeping gene 18S rRNA (catalog number: 4310893E, ThermoFisher, Waltham, MA, USA). The comparative threshold cycle method (ΔΔCt) was applied to determine the relative levels of target genes.

### 2.10 Statistical analysis

GraphPad Prism version 8.0 software was used for all statistical analyses. Parametric data were analyzed using 1-way analysis of variance (ANOVA) followed by a Tukey *post hoc* test for multiple comparisons. Nonparametric data were analyzed using the Kruskal-Wallis test for multiple comparisons or the Mann-Whitney test for two-group comparisons. A *p-*value <0.05 was considered to be significant.

## 3 Results

### 3.1 Tollip deficiency exaggerated type 2 airway inflammation in HDM-challenged and IAV-infected mice

**Figure 1:**
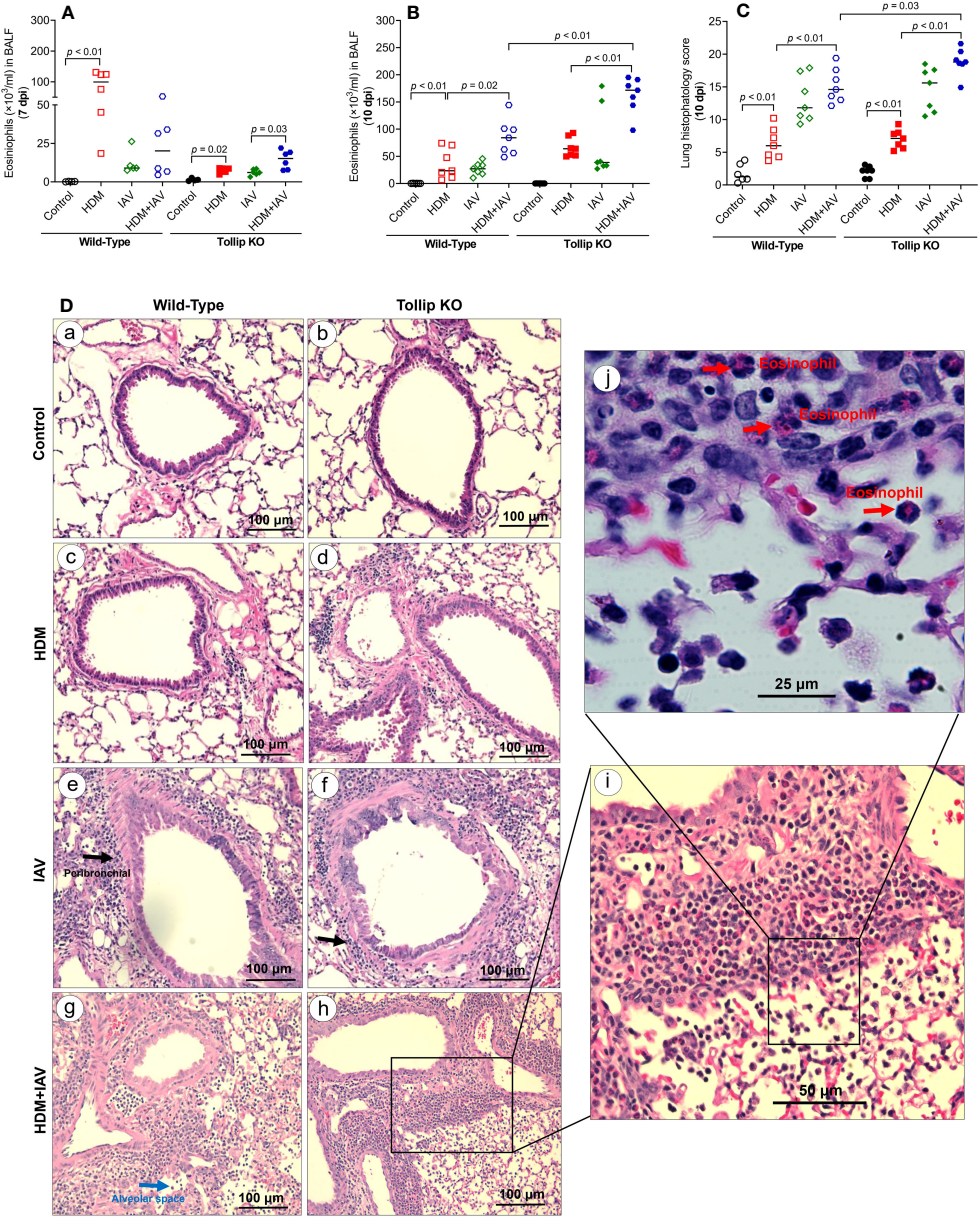
House dust mite (HDM)-challenged Tollip KO mice exaggerated lung type 2 inflammation during influenza A virus (IAV) infection. **(A, B)** Eosinophil counts in bronchoalveolar lavage fluid (BALF) of wild-type and Tollip KO mice treated with HDM and/or IAV and sacrificed at day 7 (7 dpi) and day 10 (10 dpi) after IAV infection. **(C)** Histopathology score, and **(D)** H&E staining of mouse lung tissues collected at 10 dpi. Black and blue arrows indicate leukocyte infiltration around airways and in the alveolar space, respectively. To identify inflammatory cell types (original magnification, ×200), higher magnification (×400 and ×1000), of the framed inflammation area is shown in panels (i, j). The red arrows indicate eosinophils in the submucosa and alveolar space. Data is expressed as the median of *n* = 4–7 mice/group.

In wild-type (WT) mice, HDM increased eosinophils in BALF at day 9 and 12 after the last HDM challenge (or 7 and 10 days after IAV) (**Figures 1A, B**). IAV alone in WT mice slightly increased airway eosinophils at day 7 post infection (7 dpi), which became significant at 10 dpi. While IAV infection in HDM-challenged WT mice trended to decrease eosinophil levels at 7 dpi, it significantly increased eosinophil levels at 10 dpi. After 7 days of IAV infection, Tollip deficient versus WT mice had lower levels of airway eosinophils following the treatments with HDM alone, IAV alone or combination of both HDM and IAV. However, HDM-challenged Tollip deficient mice with IAV infection trended to have higher levels of eosinophils compared to HDM or IAV alone at 7 dpi. Notably, after 10 days of viral infection, Tollip deficient mice treated with HDM and IAV had significantly higher levels of eosinophils than WT mice (**Figure 1B**).

We next examined the lung tissue histopathology to expand our findings of BALF inflammation in Tollip deficient mice at 10 dpi. The HDM-challenged Tollip KO (vs. WT) mice with IAV infection showed a significantly higher histopathology score (**Figure 1C**), indicating more severe inflammation, including eosinophils around airways and in the alveolar space (**Figure 1D**).

**Figure 2:**
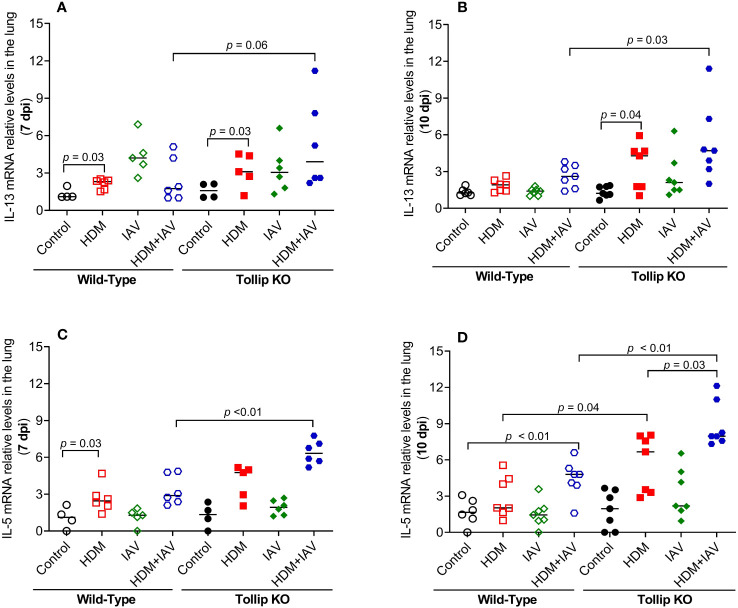
Tollip deficiency increased type 2 cytokine expression during influenza A virus (IAV) infection in house dust mite (HDM)-challenged Tollip KO mice. IL-13 **(A, B)** and IL-5 **(C, D)** mRNA expression levels in WT and Tollip KO mice sacrificed at 7 dpi (day post infection) and 10 dpi. Data is expressed as the median of *n* = 4–7 mice/group.

Eosinophil recruitment into the lung is regulated by type 2 cytokines, including IL-13 and IL-5 (35). IL-13 and IL-5 mRNA expression was increased by HDM in WT mice at 7 dpi, but not at 10 dpi. However, in the presence of both HDM and IAV infection, IL-5 increased in WT mice at 10 dpi. Tollip deficient (versus WT) mice treated with HDM and IAV significantly increased IL-13 and IL-5 mRNA expression at 7 dpi and 10 dpi (**Figures 2A–D**).

**Figure 3:**
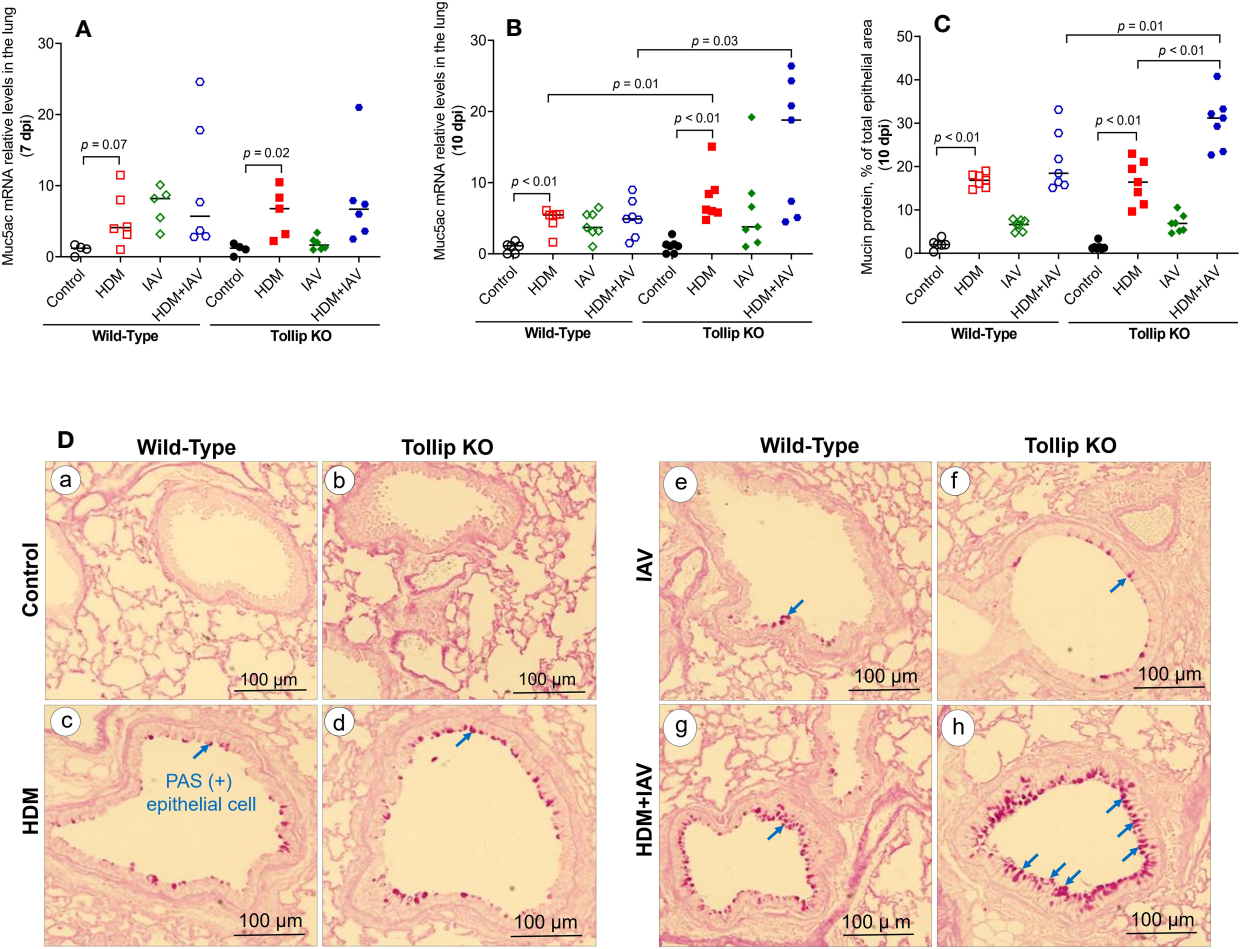
Mucin quantification in WT and Tollip KO mouse lung tissue. **(A, B)** Muc5ac mRNA expression levels in WT and Tollip KO mice at 7 dpi (day post infection) and 10 dpi. **(C)** Morphometric analysis of mucin protein expressed as a percentage of airway epithelium occupied by mucin protein. **(D)** Representative photomicrographs of airway epithelial mucin PAS staining (arrows) in control (a, b), HDM (c, d), IAV (e, f) and HDM+IAV (g, h) groups on day 10 post IAV infection. Original magnification, × 200. Data is expressed as median of *n* = 4–7 mice/group.

One of the key features of type 2 inflammation is excessive airway mucin expression or goblet cell metaplasia. As shown in **Figures 3A, B**, like the BALF eosinophil data, HDM and IAV infection increased lung tissue Muc5ac mRNA expression at 10 dpi in Tollip KO mice as compared to WT mice, which was supported by mucin protein PAS positively stained goblet cells (**Figure 3C**). Representative photomicrographs of airway mucin PAS staining were shown in **Figure 3D**.

### 3.2 Tollip-deficiency sustained airway neutrophilic inflammation in HDM-challenged and IAV-infected mice

**Figure 4:**
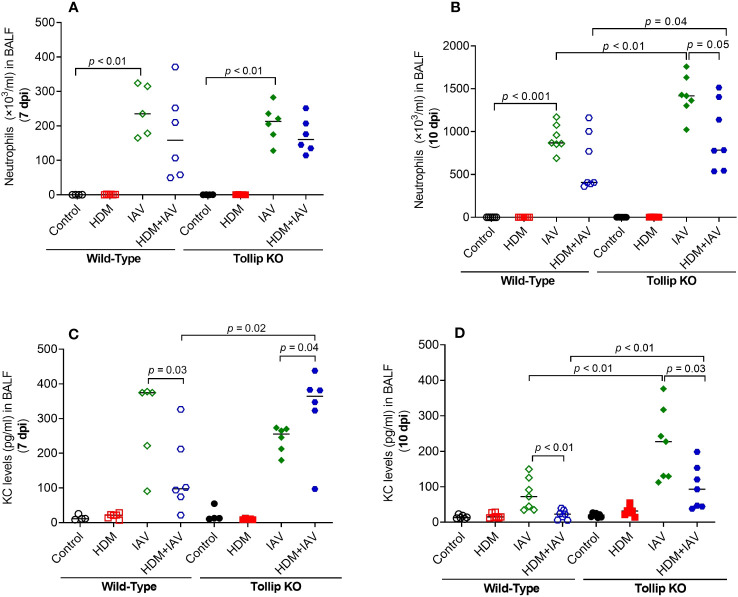
Tollip deficiency increased neutrophilic inflammation **(A, B)** and neutrophil chemokine KC **(C, D)** in WT and Tollip KO mice sacrificed at 7 dpi (day post infection) and 10 dpi. Data is expressed as the median of *n* = 4–7 mice/group.

Our previous study demonstrated that Tollip deficiency increased airway neutrophil levels after IAV infection alone (no allergen challenges) (17). Here, we extended our previous data in that Tollip deficient (vs. WT) mice treated with both HDM and IAV also had significantly higher levels of neutrophils in BALF at 10 dpi, but not at 7 dpi (**Figures 4A, B**). Interestingly, neutrophil chemokine KC preceded the increased levels of neutrophils as it was increased at 7 dpi, but not at 10 dpi in Tollip KO mice treated with HDM and IAV (**Figures 4C, D**). Unlike eosinophil and neutrophil data, the levels of macrophages and lymphocytes were not significantly different among various groups of mice.

### 3.3 Tollip deficiency promoted the release of IL-33 and blocking of IL-33 signaling inhibited type 2 inflammation

**Figure 5:**
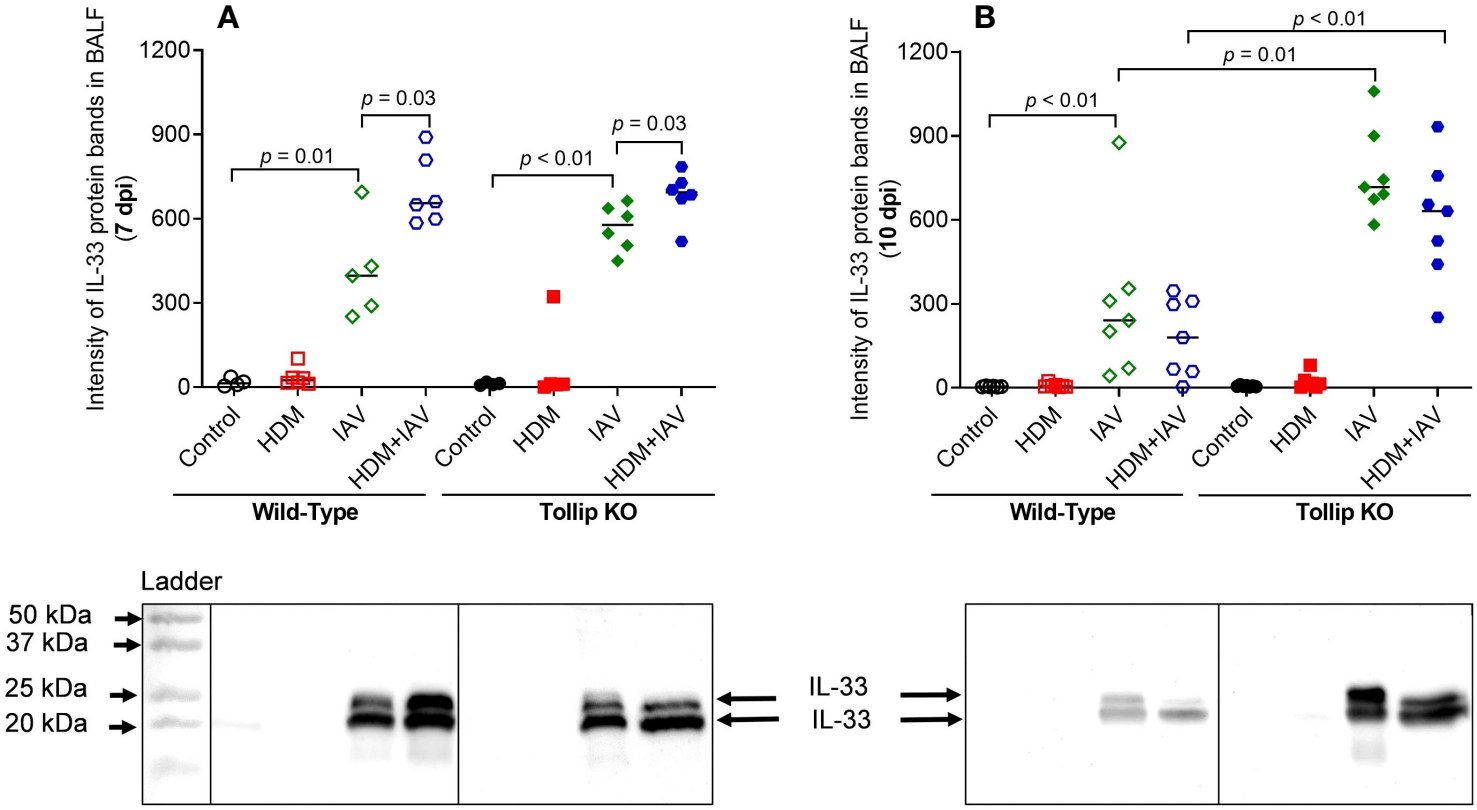
Tollip deficiency increased IL-33 release in bronchoalveolar lavage fluid (BALF). **(A, B)** IL-33 (cleaved fragments with molecular weight ≈ 20 to 25 kDa) in BALF of mice sacrificed at 7 dpi (day post infection) and 10 dpi. Western blots were normalized by loading an equal volume (25 µL) of BALF. Data is expressed as median of *n* = 4-7 mice/group.

IL-33, an upstream regulator of IL-13 *in vivo*, can be induced during viral infection (36). Given the existence of different isoforms of IL-33, including full-length (35 kDa) and active forms (18- 25 kDa), we utilized western blot to identify and quantify all isoforms of IL-33 in BALF (**Figures 5A, B**). Both 20 kDa and 25 kDa of IL-33, the fragmented (activated) isoforms derived from the full-length IL-33, were detected in BALF. Release of both 20 and 25 kDa increased similarly in WT and Tollip KO mice by IAV alone or combination of HDM and IAV at 7 dpi. However, at 10 dpi, Tollip KO mice treated with IAV and HDM + IAV had significantly higher levels of IL-33 than the WT mice.

**Figure 6:**
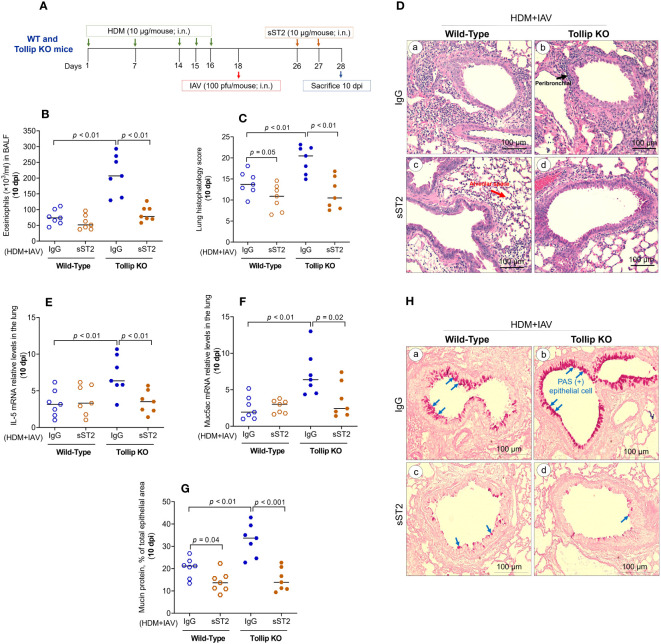
Effect of blocking IL33/ST2L signaling on lung type 2 inflammation in WT and Tollip KO mice treated with house dust mite (HDM) and infected with influenza A virus (IAV) for 10 days (10 dpi). **(A)** Study design for IL33/ST2L blocking in HDM-challenged WT and Tollip KO mice. **(B)** Eosinophils in bronchoalveolar lavage fluid (BALF). **(C)** Histopathology score, and **(D)** H&E staining of mouse lung tissues. Black and blue arrows indicate leukocyte infiltration around airways and in the alveolar space, respectively. **(E)** IL-5 mRNA levels in lung tissue. **(F)** Muc5ac mRNA levels in lung tissue. **(G)** Morphometric analysis of mucin protein, and **(H)** representative photomicrographs of airway epithelial mucin PAS staining (blue arrows) in IgG (a, b) and sST2 (c, d) treated groups on day 10 post IAV infection in HDM-challenged WT and Tollip KO mice. Original magnification, × 200. Data is expressed as median of *n* = 7 mice/group.

To determine if increased IL-33 release due to Tollip deficiency is necessary for exaggerated eosinophilic inflammation in Tollip KO mice at 10 dpi, we treated the mice with sST2 between 7 and 10 dpi (**Figure 6A**). Administration of sST2 significantly reduced airway eosinophilic inflammation (**Figure 6B**) and histopathology score (**Figure 6C**), indicating less severe inflammation around the airways and in the alveolar space (**Figure 6D**). In addition, mRNA expression of IL-5 and Muc5ac significantly decreased in Tollip KO, but not in WT mice (**Figures 6E, F**). Additionally, sST2 treatment significantly reduced PAS stained goblet cells at 10 dpi in both WT and Tollip KO mice (**Figure 6G**). Representative photomicrographs of airway mucin PAS staining after sST2 treatment were shown in **Figure 6H**.

To determine if increased IL-33 release observed in Tollip KO mice at 10 dpi was associated with lung epithelial injury, soluble E-cadherin was measured in BALF as the respiratory viral infection was shown to increase the release of E-cadherin (37). Both WT and Tollip KO mice similarly and significantly increased E-cadherin release than their non-infected control groups (**Supplementary Figure 2**), suggesting that disruption of the airway epithelial barrier may not be a mechanism for the excessive release of IL-33 due to Tollip deficiency.

### 3.4 Blocking ATP signaling inhibited IL-33 release and eosinophilic inflammation in Tollip deficient mice treated with HDM and IAV

**Figure 7:**
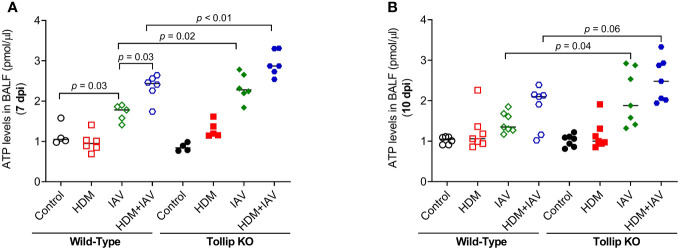
Tollip deficiency increased extracellular ATP. **(A, B)** ATP levels in bronchoalveolar lavage fluid (BALF) of WT and Tollip KO mice sacrificed at 7 dpi (day post infection) and 10 dpi. Data is expressed as the median of *n* = 4–7 mice/group.

How IL-33 is released is not well understood. Recent studies reported that IL-33 can be actively released via increased extracellular ATP (23, 27). We found that IAV infection increased ATP release into BALF in WT mice at 7 dpi, which was amplified by HDM. Tollip deficiency significantly augmented ATP release levels in response to IAV alone and combination of both HDM and IAV at 7 dpi and 10 dpi (**Figures 7A, B**). To test whether the increased ATP release is related to lung injury, lactate dehydrogenase (LDH) as an indicator of tissue injury (38) was assessed in BALF. There was no significant difference in LDH levels between WT and Tollip KO mice treated with IAV or both HDM and IAV at 7 dpi and 10 dpi (**Supplementary Figure 3**).

**Figure 8:**
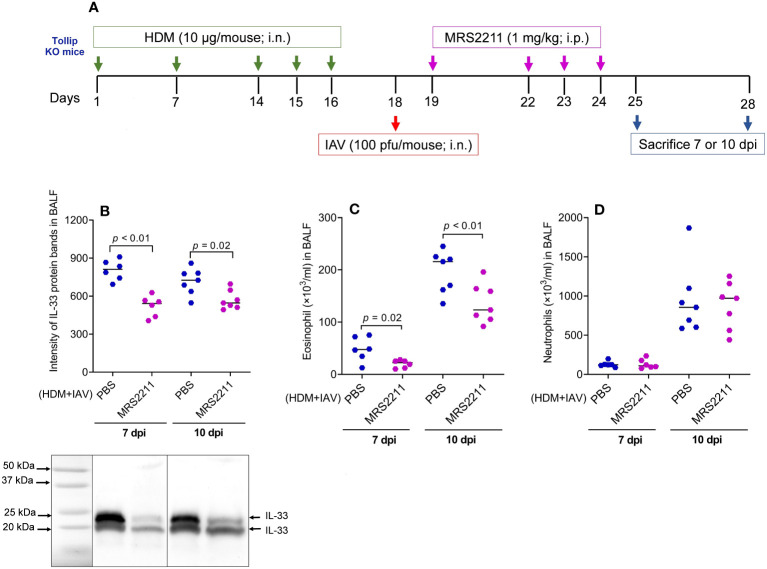
Blocking ATP receptor P2Y13 decreased IL-33 release and eosinophilic inflammation following house dust mite (HDM) challenges and influenza A virus (IAV) infection in Tollip KO mice. **(A)** Study design for P2Y13 blocking in HDM-challenged Tollip KO mice. **(B)** Western blot of IL-33 release in bronchoalveolar lavage fluid (BALF). **(C, D)** Eosinophil and neutrophil levels in BALF after MRS2211 treatment. Data is expressed as the median of *n* = 6–7 mice/group.

To determine if ATP is involved in IL-33 release and airway eosinophilic inflammation in HDM-challenged and IAV-infected Tollip KO mice, ATP signaling was blocked with MRS2211, a highly selective P2Y13 antagonist (**Figure 8A**). After MRSS2211 treatment, IL-33 release was significantly reduced at 7 dpi and 10 dpi (**Figure 8B**). Likewise, airway eosinophil, but not neutrophil recruitment was significantly attenuated by MRS2211 treatment (**Figures 8C, D**).

### 3.5 Tollip deficiency delayed the protective effect of allergen challenges on IAV infection

**Figure 9:**
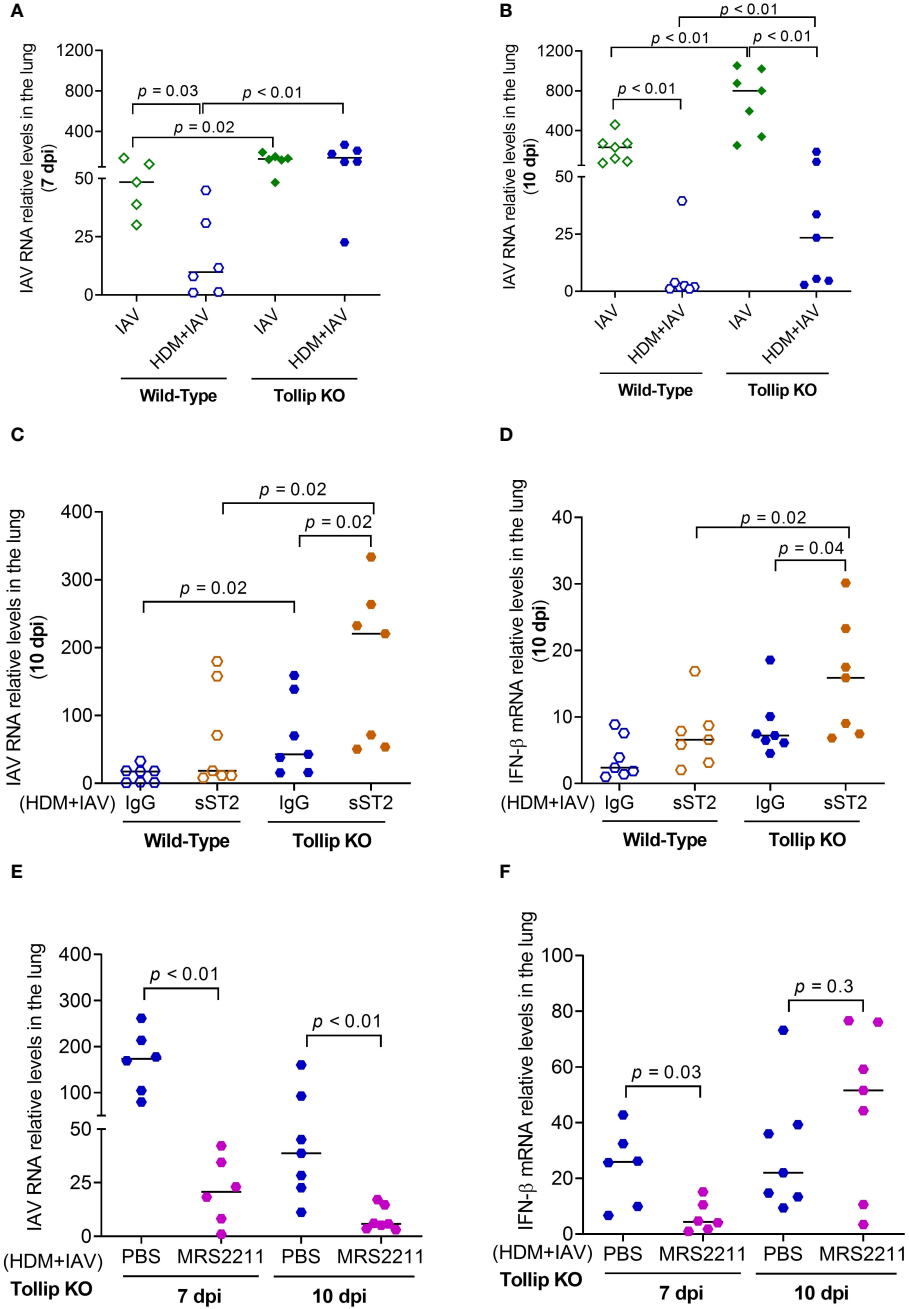
Effect of Tollip deficiency on mouse lung influenza A virus (IAV) infection. **(A, B)** Tollip deficiency increased the viral load at both 7 and 10 dpi (day post infection). Effect of IL-33/ST2L blocking with sST2 on lung tissue viral load **(C)** and IFN-β expression **(D)** in house dust mite (HDM)-challenged WT and Tollip KO mice sacrificed at 10 dpi. Effect of ATP signaling inhibition by MRS2211 on lung tissue viral load **(E)** and IFN-β expression **(F)** in HDM-challenged Tollip KO mice sacrificed at 7 dpi and 10 dpi. Data is expressed as the median of *n* = 5-7 mice/group.

The viral load in homogenized lung tissue was measured (**Figures 9A, B**). Viral load was significantly higher in Tollip KO mice than WT mice at both 7 dpi and 10 dpi in the absence of presence of HDM challenges. In WT mice, HDM significantly decreased lung viral load. Tollip deficiency either prevented (7 dpi) or slowed (10 dpi) the decline of viral load by HDM. As shown in **Figures 9C, D**, treatment with sST2 (vs. IgG control) significantly increased the viral load and IFN-β expression in Tollip KO mice at 10 dpi. In contrast, administration of MRS2211 (vs. PBS) to Tollip KO mice treated with both HDM and IAV significantly decreased lung viral load at 7- and 10 dpi (**Figure 9E**). MRS2211 treatment significantly reduced the expression of IFN-β at 7 dpi, while there was no significant change in the expression of IFN-β after 10 days of IAV infection (**Figure 9F**).

## 4 Discussion

The mechanisms of asthma exacerbations remain unclear. Our data demonstrated that Tollip deficiency may contribute to excessive lung type 2 inflammation by amplifying the release of ATP during viral infection and subsequently increasing the release of IL-33. We found that in the early stage of IAV infection, Tollip deficiency enhanced the expression of type 2 cytokines IL-13 and IL-5 in HDM-challenged mice, followed by elevated levels of lung eosinophils and airway mucin expression, another major feature of type 2 asthma. Excessive production of mucin, particularly Muc5ac, results in airway mucus plugging, persistent inflammation and airway remodeling (39). Taken together, our data suggests that Tollip deficiency may be one of the risk factors for virus-mediated exacerbations of type 2 inflammation in allergic lungs.

One innovative aspect of our current study was to unravel the role of Tollip in regulating IL-33 release during IAV infection in the presence of type 2 inflammation. For the first time, we investigated IL-33 release in HDM-challenged mice with viral infection. The role of Tollip in the release of IL-33 appeared to be time-dependent as we only observed a significant increase of IL-33 release at 10 dpi, but not 7 dpi. The current study did not determine the cellular sources of IL-33 release in BAL fluid. It is possible that multiple types of cells may contribute to the release of IL-33 as previous studies (40) found that immune cells such as eosinophils, mast cells, macrophages, dendritic cells, B cells, monocytes, and lung epithelial cells express IL-33. A critical role of IL-33 signaling in Tollip deficiency-mediated amplification of type 2 inflammation was supported by our data showing that sST2, a decoy IL-33 receptor ST2L (41), significantly reduced eosinophilic inflammation, Muc5ac and IL-5 expression. Our data are in line with previous animal studies to block IL-33 signaling (42, 43). We realize the complex role of sST2 in human asthma. High levels of sST2 in serum or induced sputum samples have shown to be predictive of asthma severity or asthma exacerbations (44–46), and were associated with high numbers of neutrophils and eosinophils. Whether sST2 plays a beneficial or detrimental role in human asthma has not been clear. Interestingly, in asthmatic children with rhinovirus infection, serum sST2 levels were reduced along with less IFN-β expression (47). In addition, cultured human airway epithelial cells reduced the release of sST2 after stimulation with a viral mimic polyI:C (48). Together, sST2 treatment may be needed to reduce excessive inflammatory responses during viral infection. However, there may be a side effect of sST2 treatment on viral infection as we have demonstrated increased viral load by administration of sST2 in Tollip deficient mice with HDM challenges.

Although IAV infection has been linked to IL-33 signaling (27), the underlying mechanisms of IL-33 release in allergic lungs remain unclear. Both passive (i.e., necrosis) and active (i.e., ATP signaling without cell death) mechanisms have been proposed to explain IL-33 release and activation (18, 49). Our data did not support cell injury as a passive mechanism of Tollip deficiency-dependent IL-33 release as levels of E-cadherin and lactate dehydrogenase were not significantly different between WT and Tollip KO mice. Another IL-33 passive release mechanism involves necroptosis which directly induces the release of nuclear IL-33 in its full-length (50). The absence of IL-33 in its full-length in BALF indicated that this process may not contribute to IL-33 release. Previous studies have shown that exposure to aeroallergens or IAV infection leads to ATP release (51, 52), which contributed to IL-33 production (27). We found that IAV infection in HDM-challenged Tollip KO mice augmented the ATP levels. Tollip is involved in the mitophagy process in order to clean the damaged mitochondria (53). Therefore, Tollip deficiency may be associated with failure to clear damaged mitochondria and increased ATP release into the cytosol or extracellular space, which may then increase the release of IL-33 from the nuclei to the extracellular space to engage in IL-33 signaling in adjacent cells. Mechanistically, extracellular ATP may activate P2Y13 on the epithelial cells and enhance IL-33 release (27). Our data demonstrated that blocking P2Y13 with MRS2211 decreased IL-33 release and lung eosinophilic inflammation in HDM-challenged Tollip KO mice. Taken together, the increased IL-33 levels in the late phase of IAV infection in HDM-challenged Tollip deficient mice may result from excessive ATP release, one of the active mechanisms of IL-33 release.

There are several interesting findings in our study. First, while increased ATP and IL-33 release is coupled with eosinophilic inflammation in HDM-challenged mice with IAV infection, the increase of eosinophils in IAV-infected mice without HDM challenges was much less despite similar IL-33 release at 10 dpi. There are several potential mechanisms to explain this observation. IAV infection is known to induce type I and type II interferons in epithelial cells and immune cells, respectively (54). Recent studies have revealed that IFN-α, -β and -γ negatively regulate production of type 2 cytokines (55–57). Furthermore, IFN-γ produced during IAV infection can inhibit IL-33-dependent type 2 inflammation (58). We found that HDM pre-exposures had a protective effect on the viral infection as viral load was reduced in wild-type mice, which was consistent with previous studies (59–62). Notably, Tollip deficiency abrogated or reduced the protective effect of HDM on viral load. Second, we observed that blocking IL-33 and ATP signaling yielded similar inhibition of type 2 inflammation, but the outcome of viral level is the opposite. While the exact mechanism remains unclear, it suggests that ATP and IL-33 may utilize differ pathways to regulate viral infection. Data from our current study and a recent publication (63) have shown the protective role of type 2 inflammation in viral infection. Thus, inhibition of type 2 inflammation by blocking IL-33 signaling (sST2 treatment) may serve as a primary mechanism for increased viral load. The fact that inhibition of ATP signaling reduced both type 2 inflammation and viral load suggests that ATP signaling may have a diverse role in viral infection. Indeed, previous studies suggest that ATP signaling (e.g., extracellular ATP levels) reflects the severity of viral infection (64) and contributes to virus production (65). Thus, it is likely that in our mouse model, ATP signaling regulates viral infection in a type 2 inflammation-independent manner. Extracellular ATP and purinergic receptors have been shown to promote the entry of HIV into target cells (66). Additionally, activation of P2Y purinergic receptors by IAV reduces alveolar fluid clearance (AFC) and causes pneumonia (67). Therefore, blocking ATP signaling may inhibit viral replication, but it should be confirmed in future studies. Therefore, inhibiting ATP signaling could be beneficial for inhibiting type 2 inflammation as well as viral infection. Lastly, how Tollip inhibits ATP and IL-33 signaling during IAV infection in HDM-challenged mice remains to be determined. Given observed mitochondrial dysfunction such as mitochondrial damage, production of excessive reactive oxygen sepsis (ROS) and ATP release in allergen exposures, inflammation and viral infection (25, 26, 68), the role of Tollip in mitochondrial function warrants further study. A previous study (28) suggests that Tollip by interacting with an E3 ubiquitin ligase Parkin is critical to coordinate the delivery of mitochondrial-derived vesicles (MDVs) to lysosomes for degradation. Therefore, it is possible that Tollip deficiency may result in impaired clearance of MDVs containing ATP synthase (69), leading to increased production of extracellular ATP. The accumulated extracellular ATP subsequently increases the release of IL-33 to enhance type 2 inflammation.

We realize some limitations to the current study. First, this study focuses on the acute model of type 2 inflammation to address the role of Tollip deficiency-mediated excessive ATP and IL-33 release in acute exacerbations of type 2-high asthma. Chronic allergen challenge models can be considered to address the long-term effect of Tollip deficiency on virus-mediated asthma exacerbations. Second, we did not test which cell types including ILC2 may produce type 2 cytokines in Tollip deficient mice with increased IL-33 release as previous studies have demonstrated an important *role of ILC2 in IL-33-mediated type 2 cytokine expression during viral infection* (70). Third, to avoid the impact of methacholine challenge on intracellular mucin levels (71–73), we did not measure airway resistance in Tollip deficient mice treated with HDM and IAV to determine if Tollip deficiency increases airway obstruction. Future experiments will be warranted to determine the effect of Tollip deficiency-mediated IL-33 release on airway obstruction. Fourth, the mechanisms by which Tollip deficiency regulates mitochondrial function such as mitophagy and ATP release need to be further explored. Fifth, we did not further explore the underlying mechanisms by which HDM exposures reduced viral load. Previous studies suggested that eosinophils may protect against viral infection (74). As HDM challenges increased lung eosinophils, it is conceivable that viral load would be decreased. As there are multiple mechanisms involved in viral defense or clearance, therapeutic approaches to target type 2 inflammation should be carefully balanced with consideration of viral infection. Finally, although we focused on the measurement of neutrophil chemokine KC, there may be other cytokines (e.g., IL-17) contributing to neutrophilic inflammation. We measured IL-17 mRNA expression in IAV-infected lung tissue at 10 dpi. Overall, IAV-infected Tollip KO mice showed higher levels of IL-17 mRNA expression. However, unlike KC data, IL-17 mRNA expression did not differ between the IAV and IAV+HDM groups in both wild-type and Tollip KO mice. Therefore, our data suggest that KC may better indicate the changes of neutrophilic inflammation in our mouse model.

**Figure 10:**
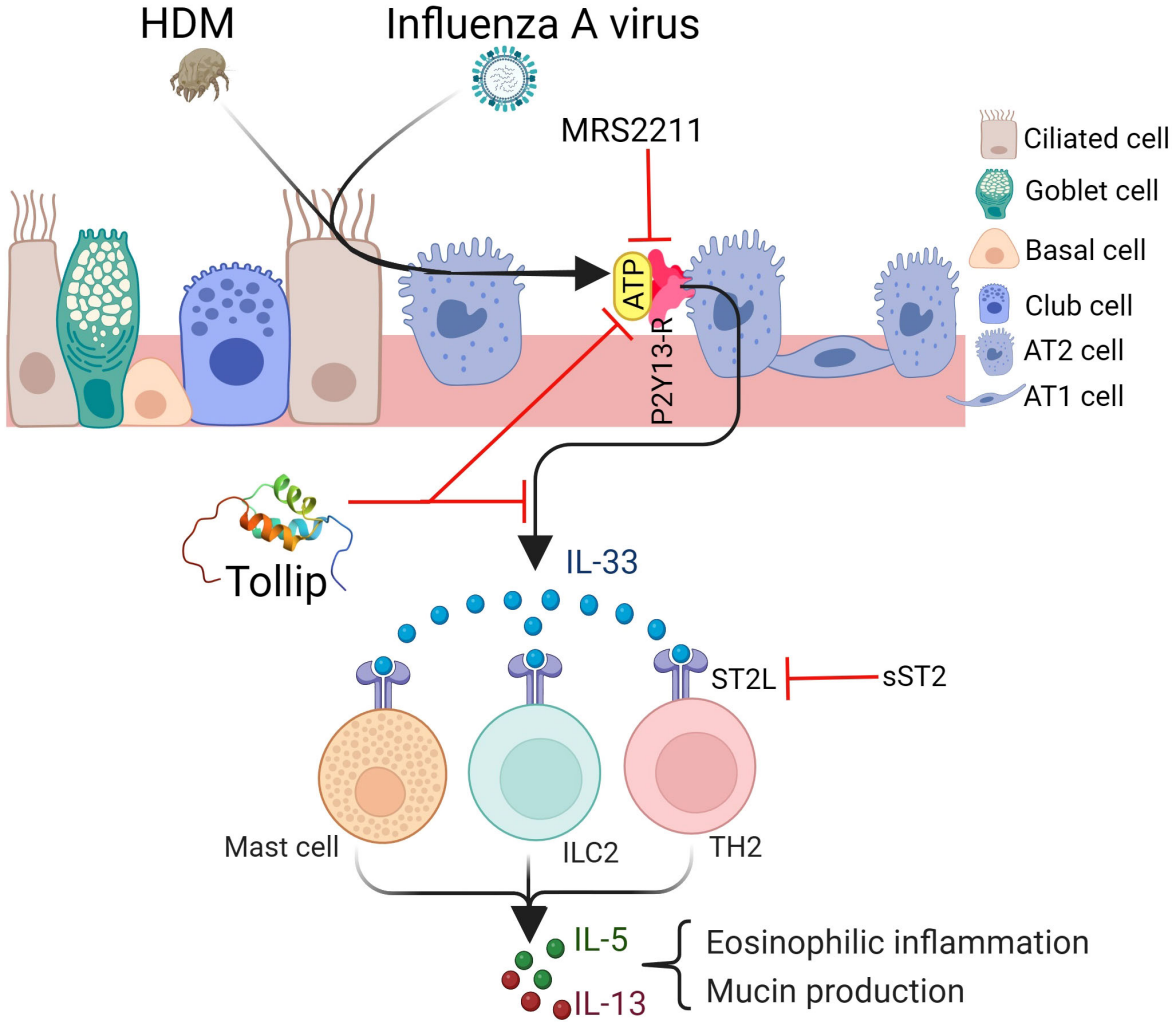
Proposed mechanisms by which Tollip regulates type 2 inflammation during influenza A virus (IAV) infection. Tollip deficiency enhances the release of ATP in lungs exposed to allergens and viruses. ATP binds to its receptor P2Y13 on epithelial cells, leading to IL-33 release. IL-33 binds to ST2L on different cell types, and results in type 2 cytokine release. Blocking ATP and IL-33 signaling by MRS2211 and sST2 attenuates excessive type 2 inflammation during IAV infection in Tollip deficient lungs. AT1; alveolar type I cell, AT2; alveolar type II cell. Created with BioRender.com. Tollip structure was extracted from protein data bank (www.rcsb.org).

In summary, our study, for the first time, demonstrated that Tollip deficiency serves as a risk factor for exaggerating type 2 inflammation during IAV infection, potentially through the ATP/IL-33 signaling axis (**Figure 10**). Our findings have the potential therapeutic implication of using the ATP receptor antagonists to treat excessive type 2 inflammation in patients with Tollip deficiency who experience exacerbations associated with IAV or other species of viral infections.

## Data availability statement

The raw data supporting the conclusions of this article will be made available by the authors, without undue reservation.

## Ethics statement

The animal study was approved by Animal Care and Use Committee at National Jewish Health. The study was conducted in accordance with the local legislation and institutional requirements.

## Author contributions

HN: Data curation, Investigation, Methodology, Writing – original draft, Writing – review & editing. NS: Methodology, Writing – review & editing. MK: Conceptualization, Investigation, Methodology, Writing – review & editing. LL: Methodology, Writing – review & editing. MN: Investigation, Methodology, Writing – review & editing. HC: Conceptualization, Data curation, Funding acquisition, Investigation, Project administration, Resources, Supervision, Validation, Visualization, Writing – review & editing.
