# Supplementary material for: Tollip deficiency exaggerates airway type 2 inflammation in mice exposed to allergen and influenza A virus: role of the ATP/IL-33 signaling axis
Source: Front Immunol. 2023 Dec 6;14:1304758. doi: 10.3389/fimmu.2023.1304758 (PMC10731025; doi:10.3389/fimmu.2023.1304758)
Supplement: Supplementary file 1 [file DataSheet_1.docx]

Supplementary Material

**Tollip deficiency exaggerates airway type 2 inflammation in mice exposed to allergen and influenza A virus infection: Role of the ATP/IL-33 signaling axis**

**Hamid Reza Nouri, Niccolette Schaunaman, Monica Kraft, Liwu Li, Mari Numata, and Hong Wei Chu^*^**

^*^ **Corresponding author:**

Hong Wei Chu, M.D., Department of Medicine, National Jewish Health, 1400 Jackson St., Room A639, Denver, CO 80206, USA. Phone: 303-398-1689; E-mail: [chuhw@njhealth.org](mailto:chuhw@njhealth.org)

**
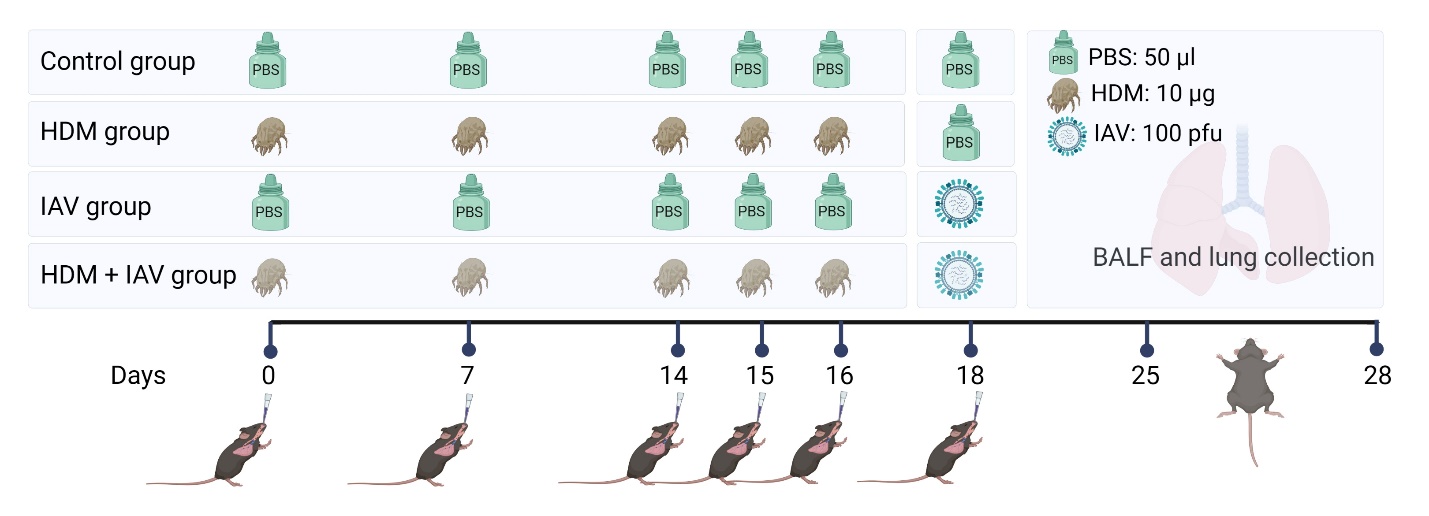
**

**Figure S1.** Experimental design. Tollip knockout (KO) and wild-type (WT) mice were intranasally challenged with 10 μg of house dust mite extract (HDM) on days (0, 7, 14, 15 and 16), and were subsequently (day 18) inoculated with 1×10^2^ PFU/mouse of influenza A virus (IAV). Control group of mice was pretreated with phosphate-buffered saline (PBS) and subsequently administered PBS (control) or inoculated with IAV (IAV group). Mice were sacrificed at 7- and 10-days post IAV infection to collect bronchoalveolar lavage fluid (BALF) and lung tissue for cellular and molecular evaluation. Created with BioRender.com.


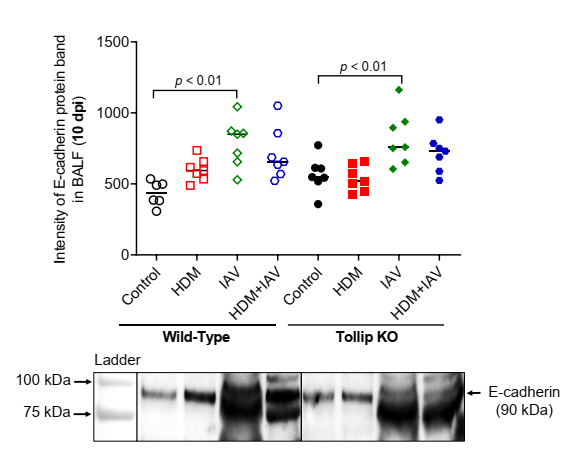


**Figure S2.** IAV infection increased the soluble E-cadherin levels in BAL fluid of WT and Tollip KO mice. A representative western blot of E-cadherin protein is shown below the densitometric data. Data are expressed as median of *n* = 6–7 mice/group.


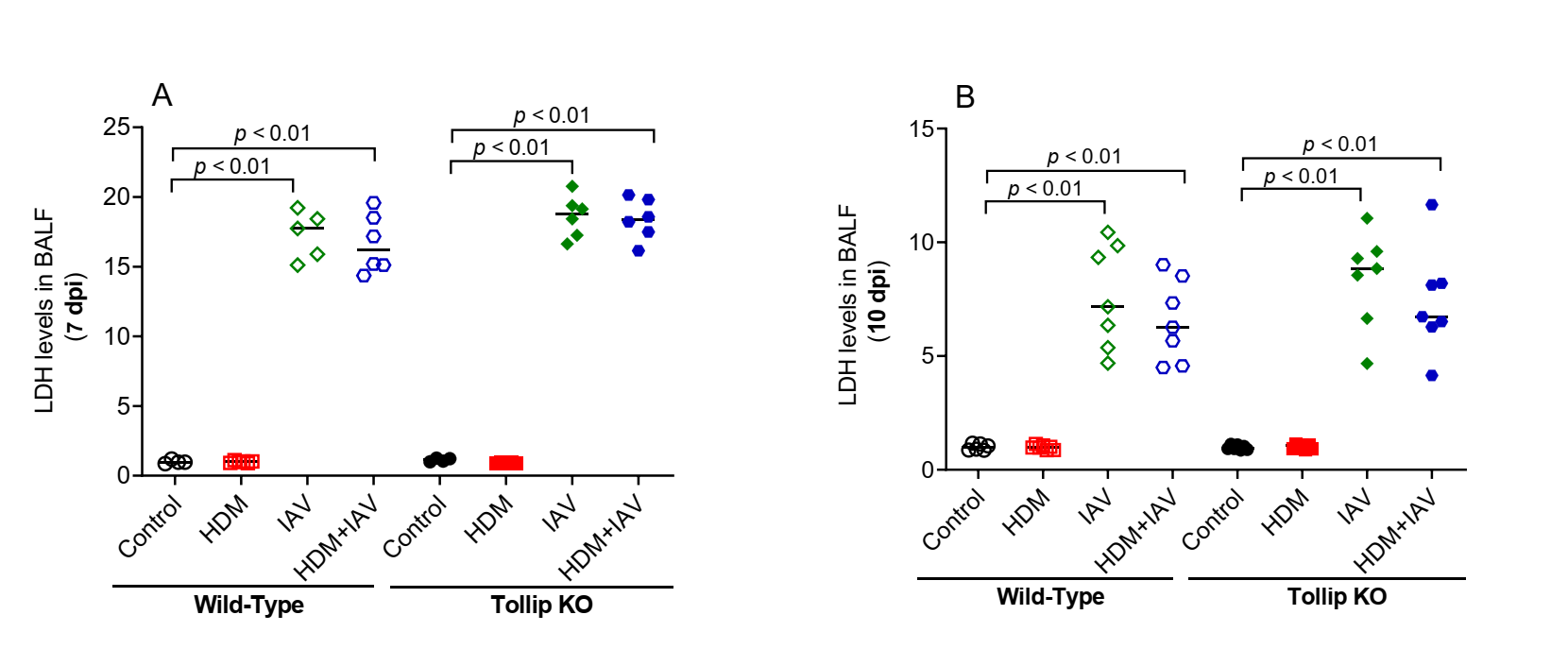


**Figure S3**. Cytotoxicity was analyzed by performing the LDH assay. (A) LDH level was increased during IAV infection on 7 dpi and (B) 10 dpi, but there was no significant difference between WT and Tollip KO mice. Data are expressed as median of *n* = 4–7 mice/group.
